# T3 versus T4 video-assisted thoracoscopic sympathectomy for palmar hyperhidrosis: A protocol for a systematic review and meta-analysis

**DOI:** 10.1097/MD.0000000000017272

**Published:** 2019-10-18

**Authors:** Sui Chen, Peipei Zhang, Tianci Chai, Zhimin Shen, Mingqiang Kang, Jiangbo Lin

**Affiliations:** a Department of Thoracic Surgery, Fujian Medical University Union Hospital, Fuzhou, China; b Department of anesthesiology, Xinyi People's Hospital, Xuzhou, China.

**Keywords:** meta-analysis, palmar hyperhidrosis, video-assisted thoracoscopic sympathectomy

## Abstract

**Background:**

Palmar hyperhidrosis (PH) is a common sympathetic disorder that reduces patient’ quality of life. Video-assisted thoracoscopic sympathectomy (VTS) is a popular and effective treatment for PH. However, there is substantial controversy about the treatment of PH with VTS at the T3 or T4 level. We will compare the quality metrics of VTS at T3 versus T4 to determine the optimal level for VTS.

**Methods:**

We will search PubMed, Scopus, Web of Science, Embase, Cancerlit, the Cochrane Central Register of Controlled Trials, and the Google Scholar databases for relevant clinical trials published in any language before March 31, 2019. Randomized controlled trials (RCTs), quasi-RCTs, propensity score-matched comparative studies, and prospective cohort studies of interest, published or unpublished, that meet the inclusion criteria will be included. Subgroup analysis of the type of operation, sex of patient, and ethnicity of patient will be performed.

**Results:**

The results of this study will be published in a peer-reviewed journal.

**Conclusions:**

The results of this study will provide reliable evidence for the development of optimal treatment strategies for patients with PH. Owing to the characteristics of disease and intervention methods, randomized controlled trials may not be sufficient. We will include high-quality nonrandomized controlled trials, but this may lead to high heterogeneity and may affect the reliability of the results.

**PROSPERO registration number:**

CRD42018116607.

## 1 Introduction

palmar hyperhidrosis (PH) is a common symptom of sweat gland hypersecretion caused by sympathetic nerve dysfunction, which seriously adversely affects patient social and quality of life.^[1]^ The sympathetic nerve that controls sweat glands in the hand comes from the second thoracic ganglion (T2) ∼fifth thoracic ganglion (T5), and most of the control comes from the T2. The preganglionic fibers of the arm mainly originate from the third to sixth segments of the spine.^[2]^ Early thoracic sympathectomy is performed at the level of T2,^[3–6]^ but the postoperative complications including hand trunk, compensatory hyperhidrosis and other complications have increased significantly.^[7,8]^ Currently, video-assisted thoracoscopic sympathectomy (VTS) at the T3 or T4 level is widely performed for the treatment of PH worldwide with a better effectiveness and safety profile than at the T2 level,^[9–14]^ however, the optimal level in which to perform VTS remains controversial. Excessive high-level VTS may lead to dry hands and compensatory hyperhidrosis, but low-level VTS may result in a poor surgical outcome.^[15–20]^ To determine the optimal level of VTS, we will conduct a systematic review and meta-analysis of published or unpublished related studies.

## 2 Objective

A meta-analysis and systematic review will be conducted to compare the efficacy and safty of T3 versus T4 VTS for PH.

## 3 Methods

This protocol was designed to adhere to the Preferred Reporting Items for Systematic Review and Meta-Analysis Protocols (PRISMA-P) statement.^[21]^ The results of this study will be reported according to the Preferred Reporting Items for Systematic Reviews and Meta-Analysis (PRISMA) guidelines.^[22]^

### 3.1 Patient and public involvement

This study will be based on published studies, unpublished studies, and records and will not directly involve patients or the public.

### 3.2 Eligibility criteria

#### 3.2.1 Types of studies

We will include published or unpublished randomized controlled trials (RCTs) or quasi-RCTs, propensity score-matched comparative studies, and prospective cohort studies.

#### 3.2.2 Types of participants

The participants included will be patients diagnosed with PG that were treated with VTS at the T3 or T4 levels. No restrictions regarding sex, ethnicity, economic status, and education will be applied.

#### 3.2.3 Types of interventions

All forms of VTS at the T3 level were compared with VTS at the T4 level for the treatment of PH.

#### 3.2.4 Types of outcome measures

##### 3.2.4.1 Primary outcomes

We will assess symptom resolution and the satisfaction of patients with PH after treatment with 2 different levels of VTS. This will be reported as the symptom resolution rate and the degree of patient satisfaction.

##### 3.2.4.2 Secondary outcomes

Compensatory hyperhidrosis, dry hands, and gustatory sweating, all common postoperative complications associated with the level of VTS and other postoperative complications associated with VTS, will be included. We will assess these complications and the recrudescence of PH. This will be reported as the complication rate and recurrence rate.

### 3.3 Information sources

We will search Embase, Scopus, Pubmed (Medline), Google Scholar, and the Cochrane Central Register of Controlled Trials for related studies published in any language before March 31, 2019.

### 3.4 Search strategy

We will search the electronic databases mentioned above for eligible studies without any language restrictions. The relevant keywords of the search are related to Medical Subject Heading (MeSH) terms. The search strategies for PubMed are shown in Table 1.

**Table 1:**
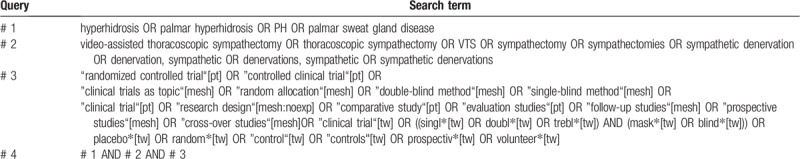
PubMed search strategies.

### 3.5 Data collection and analysis

We will summarize the evidence using the methods described in the Cochrane Handbook for Systematic Reviews of Interventions.^[23]^

### 3.6 Study selection

Two review authors (SC, PPZ) will independently screen titles and abstracts of all studies searched and exclude those which do not meet the inclusion criteria. The reasons for exclusion will be documented by SC and PPZ. The full text of all possible eligible studies will be retrieved and the 2 authors (SC, PPZ) will independently assess the eligibility of the retrieved articles. We will resolve disagreements between the 2 reviewers by discussion. If necessary, we will consult the third review author (MQK). The selection process will be shown in the PRISMA flow chart in detail.

#### 3.6.1 Data extraction and management

The following data will be extracted independently by two review authors (SC, PPZ) from the included studies: study characteristics and methodology (the first author, publication date, country, study design, randomization, periods of data collection, total duration of study, follow-up duration, withdrawals, among others); participants (sex, age, weight, height, classification of PH, inclusion and exclusion criteria, among others); interventions (the level of VTS, manners of sympathectomy, location of surgical incision, duration, thoracic drainage, among others); outcomes and other data (symptom resolution, satisfaction of patients, length of hospitalization, compensatory hyperhidrosis, dry hands, gustatory sweating, recurrence rate, other postoperative complications, among others). There will be no difference in data extraction for different sympathectomy techniques. We will extract all of the relevant data and record it in a predesigned table. If the reported research data are unclear or missing, we will consult the authors by e-mail to determine whether the relevant data will be included.

### 3.7 Assessment of risk of bias in the included studies

Two review authors (SC, PPZ) will evaluate the risk of bias of each study independently using the Cochrane Handbook for Systematic Reviews. They will assess the risk of bias independently according to the following domains: random sequence generation (selection bias), allocation concealment (selection bias), blinding of participants and personnel (performance bias), blinding of outcome assessment (detection bias), incomplete outcome data (attrition bias), selective outcome reporting (reporting bias), and other bias. They will assess each domain independently and report as high, low, or uncertain risk of bias, and the assessment results will be recorded in the risk of bias table in detail.

### 3.8 Data analysis

Review Manager 5.3 software will be used to process and analyze the data gathered from the included clinical trials. We will use the Cochran *Q* and *Ι*^2^ statistic to assess heterogeneity among the studies versus matched pairs for the standard meta-analysis. If there is obvious heterogeneity (*χ*^2^ or *I*^2^ statistic >50%), the trials will be judged to have high heterogeneity and we will use a random-effects model to analyze the data. Otherwise, the fixed-effect model will be adopted to analyze the data. We will utilize the Mantel-Haenszel method to pool the binary data. We will report the results in the form of relative risk within the 95% confidence interval (CI) of the data. We will utilize the inverse variance analysis method to pool continuous data and the results will be shown in the form of standardized mean difference within the 95% confidence interval (CI) of the data.

#### 3.8.1 Subgroup analysis

If there is obvious heterogeneity, and the available data are sufficient, then we will conduct subgroup analysis to search for possible causes of heterogeneity. If available data are sufficient, then we will conduct subgroup analysis according to the grading of symptoms, patient sex, and region.

#### 3.8.2 Sensitivity analysis

We will conduct sensitivity analysis to evaluate the robustness and reliability of the aggregation results by excluding trials with a high risk of bias.

### 3.9 Publication bias

If there are at least 10 studies included, we will utilize a funnel plot and Egger test to investigate publication bias. If publication bias is suspected, we will contact the study investigators to obtain more information. If bias does exist, the use the fill and trim method to analyze reporting bias in the trials.^[24]^

### 3.10 Evidence evaluation

All evidence will be evaluated by the criteria of GRADE (study limitations, imprecision, consistency of effect, publication and indirectness bias) and the quality of overall evidence will be judged at 4 levels (high, moderate, low, and very low).^[25]^

## 4 Discussion

PH is a symptom of excessive secretion of the hand sweat glands caused by sympathetic nerve dysfunction that controls hand sweat glands. It has a negative effect on the quality of life of PH patients. The routine medical treatment of PH cannot achieve the desired outcome. Thoracic sympathectomy is the most effective treatment for PH. With further study and better understanding of PH, traditional T2 level thoracic sympathectomy, which only has the goal of symptom resolution but has a high risk of postoperative complications (such as compensatory hyperhidrosis and dry hands) can be replaced by T3 or T4 level VTS with fewer complications and better outcomes.^[26]^

VTS is the preferred surgical treatment for PH, but VTS at different levels has different outcomes after surgery. However, VTS at level T3 or T4 remains controversial. Therefore, we will conduct a systematic review and meta-analysis of published or unpublished related clinical studies to determine the optimal level of VTS and to provide guidance for clinical surgeons in choosing the best surgical approach.

## Author contributions

**Conceptualization:** Sui Chen, Peipei Zhang, Tianci Chai.

**Data curation:** Sui Chen, Peipei Zhang, Tianci Chai, Zhimin Shen.

**Formal analysis:** Sui Chen, Peipei Zhang, Tianci Chai, Zhimin Shen, Mingqiang Kang.

**Funding acquisition:** Mingqiang Kang.

**Investigation:** Sui Chen, Peipei Zhang.

**Methodology:** Sui Chen, Peipei Zhang.

**Project administration:** Sui Chen, Peipei Zhang, Mingqiang Kang.

**Resources:** Sui Chen, Peipei Zhang.

**Software:** Sui Chen.

**Supervision:** Sui Chen.

**Validation:** Sui Chen.

**Visualization:** Sui Chen.

**Writing – original draft:** Sui Chen.

**Writing – review & editing:** Mingqiang Kang.
